# The New Orleans Meeting of the American Medical Association

**Published:** 1885-03

**Authors:** 


### The New Orleans Meeting of the American Medical Association.

The distinguished President, Dr. Henry F. Campbell, of Augusta, Ga., is entitled to the full support of the profession in the way of attendance and papers at the approaching meeting of the National Medical Association, which will be held in the city of New Orleans, commencing Tuesday, April 28, 1885, and continuing four days. Every American physician should take pride in this body, and it is to be hoped that the meeting will be successful, and in every way a credit to us as a progressive profession. In this connection we notice that the Illinois Central Railroad will run a special excusion train of Pullman cars from Chicago to New Orleans, leaving at 9 P. M., April 25th, making the trip in thirty-six hours. The prospects for a pleasant trip are thus very favorable, and members going by this route will be enabled to return via Mobile, Montgomery, Nashville, Mammoth Cave, etc., over the Louisville and Nashville line, or via Meridian, Chattanooga and Cincinnati, over the Cincinnati, New

Orleans and Texas line. Round-trip tickets will be issued and can be purchased in Chicago for any of these routes which may be selected at the time of purchase. The rates will be very low and tickets good for thirty days, with stop-over privileges and a reduction of sleeping-car fare. We hope some arrangement may be made of a similar nature from this city, to enable delegates from this region to enjoy equally fair advantages with the Chicagoians.

---

One of the most ingenious ventures in professional advertising which we have noticed of late, will be found in a Sunday morning paper of late date. The specialists succeed admirably in evading the Code, new and old, and also have little of that delicate sense of propriety prevailing among members of the profession. They make some excuse to give the dear public full notice of their professional accomplishments, and also of their contributions to the medical literature of the day, and thus "whip the devil around the stump," with the end in view, evidently, of personal popularity. We have witnessed this tendency among specialists frequently, and we are led to congratulate ourselves that the broader specialty of *general practice* antagonizes the egotism engendered by too close attention to a part rather than the whole of the human organism, and thus saves the chagrin which must follow the adoption of such subterfuges for the attainment of fame and reputation. The spirit of the Code of Ethics is opposed to methods such as we criticise. Unless followed, what is the use of it? It is too often a dead-letter on the statute-book.

---

The following advertisment was placed in the hands of one of the prominent printing houses of this city for publication in the form of a circular, for general distribution. We print it as it was written, without changing its orthography, the construction of its sentences or capitalization of words. May it not in truth be said that there is need of higher education among medical men, or the necessity of a State Board of Examiners for license to practice? The latter measure would exterminate from the State the army of quacks and mountebanks who prey upon the credulity of the people.

GREAT REWARD OFFARD.

To Dr. Jas. H. Benson formaly of New York the Reward of Merrit for the instant releaf and permant cure of Putrid Soar Throat, this Medisan is sold by all Drugasts. inquire for Dr. Jas. H. Benson Throat Powder and gargle directions for uce on Evrey package, Price 75 cts. he would Alsow Stait to parties in Buffalo that ar Troubled with the folowing Deseases Sutch as Chronick Rumatism, Chronick Direah, Cansers, Femail Complaints, Asmay, Consumsion and all of Seated Deseases to call at 414 Michigan st. free Examinations Givon.

